# Antihypertensive medication adherence and associated factors among adult hypertensive patients at Jimma University Specialized Hospital, southwest Ethiopia

**DOI:** 10.1186/s13104-018-3139-6

**Published:** 2018-01-15

**Authors:** Solomon Weldegebreal Asgedom, Tesfay Mehari Atey, Tigestu Alemu Desse

**Affiliations:** 1 School of Pharmacy, College of Health Sciences, Mekelle University, Mekelle, Ethiopia; 2 Department of Pharmacology and Clinical Pharmacy, School of Pharmacy, College of Health Sciences, Addis Ababa University, Addis Ababa, Ethiopia

**Keywords:** Adherence, Antihypertensive medications, Jimma University Specialized Hospital

## Abstract

**Background:**

Adherence to antihypertensive medications is a key component to control blood pressure levels. Poor adherence to these medications leads to the development of hypertensive complications and increase risk of cardiovascular events which in turn reduces the ultimate clinical outcome. The purpose of this study was to assess antihypertensive medication adherence and associated factors among adult hypertensive patients. A hospital-based cross-sectional study among adult hypertensive patients was conducted at hypertensive follow-up clinic of Jimma University Specialized Hospital from March 4, 2015 to April 3, 2015. A simple random sampling technique was used to select the study participants from the study population. The study patients were interviewed and their medical charts were reviewed using a pretested structured questionnaire. Adherence was assessed using Morisky Medication Adherence Scale-8 (MMAS-8) and MMAS-8 score less than 6 was considered as non-adherent and MMAS-8 score was ≥ 6 was declared as adherence. Factors associated with adherence were identified using binary and multivariate logistic regression analysis. Crude odds ratio, adjusted odds ratio (AOR) and 95% confidence interval of the odds ratio were calculated using SPSS version 21. Variables with *p*-value less than 0.05 were assumed as statistically significant factors.

**Results:**

Among 280 hypertensive patients, 61.8% of the study participants were found to be adherent. More than half (53.2%) of the participants were males and the mean age of the participants was 55.0 ± 12.7 years. Co-morbidity (AOR = 0.083, 95% CI = 0.033–0.207, *p* < 0.001), alcohol intake (AOR = 0.011, 95% CI = 0.002–0.079, *p* < 0.001), getting medications freely (AOR = 0.020, 95% CI = 0.003–0.117, *p* < 0.001), and combination of antihypertensive medications (AOR = 0.32, 95% CI = 0.144–0.712, *p* < 0.005) were inversely associated with antihypertensive medication adherence.

**Conclusion:**

The adherence level to the prescribed antihypertensive medications was found to be sub-optimal according to the MMAS-8, and influenced by co morbidity, alcohol intake, self-purchasing of the medications and combination of antihypertensive medications.

**Electronic supplementary material:**

The online version of this article (10.1186/s13104-018-3139-6) contains supplementary material, which is available to authorized users.

## Background

Hypertension is the most significant risk factor for cardiovascular disease and its prevalence is still increasing globally [1–3]. Worldwide, more than half of the hypertensive patients have systolic blood pressure (SBP) ≥ 140 and/or diastolic blood pressure (DBP) ≥ 90 mmHg [4]. The rate of blood pressure (BP) control varies from country to country in which the level of controlled BP among treated cases were 65, 50, 40, 32.8 and 30% in Bahrain, USA, England, Zimbabwe, and Germany respectively [5–9]. In developing countries, the high prevalence of hypertension compounded with poor hypertension control remain a significant public health problem [10–12].

Taking antihypertensive medications properly is a central point in the management of hypertension. Effective antihypertensive treatment should be maintained indefinitely to reduce the relative risk of stroke and other cardiovascular disease events [13]. However, due to the asymptomatic nature of the disease and indefinite treatment duration, medication adherence remains a significant challenge among these patients. Therefore, adherence to antihypertensive medication therapy is the main predictor of treatment success and an effective step in controlling BP and preventing complications [14].

The World Health Organization (WHO) identifies poor adherence as the most significant cause of uncontrolled BP and estimates that 50–70% of people do not take their antihypertensive medication as prescribed [15]. In Ethiopia, studies done in Adama and Gonder showed that the level of adherence to antihypertensive treatment was 59.9 and 64.6% respectively. The level of adherence in the studies meant that percentage of patients having good adherence divided by the total studied population [16, 17].

Poor adherence is responsible for unnecessary over-prescription of drugs, substantial worsening of diseases, increases in avoidable hospital admission rates and longer hospital stays which all leading to a significant medical burden such as reduced optimal clinical benefit and increased risk of cardiovascular events [14, 18–20]. Additionally, findings in clinical practice have raised issues about under treatment and non-adherence to antihypertensive treatment hampering the effectiveness of the medications [21].

There are various factors that affect a hypertensive patient’s behavior regarding adherence to antihypertensive treatments. Knowledge about hypertension and its treatment, socio-demographics, beliefs about treatment, patient-provider relationship and the support received from healthcare services are the factors that affect hypertensive patient’s adherence [22]. Identifying factors that affect medication adherence is the first step towards improving adherence [23]. Therefore; the aim of this study was to assess antihypertensive medication adherence and associated factors among adult hypertensive patients attending Jimma University Specialized Hospital (JUSH).

## Methods

A hospital-based cross-sectional study among hypertensive adult patients was conducted at the hypertension clinic of JUSH from March 4, 2015 to April 3, 2015. JUSH is a teaching and referral hospital with 523 beds serving for approximately 20,000 admissions and 140,000 outpatient visits a year with a catchment population of about 15 million people. Hypertensive patients receive services and medications every month at the clinic. Considering 1.96 for the standard normal variable with 5% level of significance (α-value), 95% confidence interval, 5% margin of error and 10% contingency, the sample size was calculated to be 311 from the study population.

The study was approved by the Institutional Review Board of Collage of Public Health and Medical Sciences, Jimma University. An informed written consent was obtained from all patients. Then face-to-face interview with a pretested structured questionnaire was conducted to collect the socio-demographics, clinical characteristics and adherence status of patients to antihypertensive medication(s). The face to face interview was employed using six trained clinical nurses. The data were collected by a trained six clinical nurses who used to work at the hypertensive clinic but who did not work at the clinic during the study period. Data on co-morbidities, antihypertensive medication(s) and a 1 year BP measurements were extracted from medical records of patients using a pretested data abstraction format.

All hypertensive patients aged 18 years and above, who had a regular follow-up for at least 12 months at the clinic, patients who used an antihypertensive medication for hypertension and whose medical records contained complete data and who were willing to participate were included in the study. Seriously ill patients who were not able to finish the interview, patients on DASH therapy alone (i.e. with no drug therapy) and patients with incomplete medical records were excluded from the study.

To maintain validity of the data collection tool, a structured questionnaire was developed and translated into local languages (Amharic and Afan Oromo) and back translated to English. An eight item Morisky’s Medication Adherence Scale (MMAS-8) was employed to assess the study participant’s medication adherence. A scoring scheme of “Yes” = 0 and “No” = 1 for the first seven items except item number five in which the values are of “Yes” and “No” were reversed and for the last item a five-point Likert response was used with options “never”, “once in a while”, “sometimes”, “usually”, and “always” (Additional file 1) [24]. The primary outcome of the study was antihypertensive medication adherence status.

### Operational definitions

Hypertension was defined as sustained high BP (SBP ≥ 140 or DBP ≥ 90 mmHg) or reported regular use of antihypertensive medication(s) [9]. Uncontrolled BP was defined as SBP of ≥ 140 mmHg and/or DBP of ≥ 90 mmHg [25]. Controlled BP was defined as SBP of < 140 mmHg and/or DBP of < 90 mmHg [25]. Patients were considered as non-adherent to their medication(s) when MMAS-8 score was less than 6 and adherence was considered when the patient’s MMAS-8 score was ≥ 6 [26, 27]. Rate of adherence was defined as the number of adherent participants divided by the total number of participants. Rate of BP control was also defined as the number of participants with controlled BP divided by the total number of participants.

### Statistical analysis

Data were analyzed using Statistical Package for Social Sciences (SPSS^®^statistics) program version 21 (SPSS; Chicago, IL, USA). Binary logistic regression analysis was employed to identify determinants of medication adherence. Variables with *p*-values < 0.25 on a univariate logistic regression model were entered into a multivariate logistic regression and variables with *p* value < 0.05 were considered as statistically significant.

## Results

**Fig. 1:**
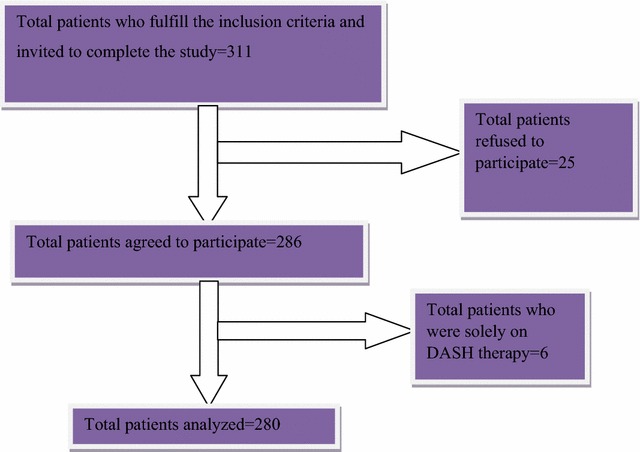
Hypertension patient selection flow chart at Jimma University Specialized Hospital from March 4, 2015 to April 3, 2015. *DASH* dietary approach to stop hypertension

In this study, a total of 311 participants were fulfilled inclusion criteria and invited to interview and of this, 286 had completed interview. Of the 286 participants who completed interview, 6 participants were on a DASH therapy and were excluded from analysis (Fig. 1). The overall response rate was 92%. As shown in Table 1, more than half (53.2%) of the participants were males. The mean age of the participants was 55.0 ± 12.7 years, with minimum 26 and maximum of 94 years old. About one-fourth (n = 25.4%) of the patients were elders (65 years and older). Above three-quarter (78.6%) of the participants were married. Regarding duration of hypertension, 162 (57.9%) patients have a duration of less than 5 years (Table 1).

**Table 1:** Frequency distribution of socio demographic and clinical characteristics of hypertensive patients at Jimma University Specialized Hospital from March 4, 2015 to April 3, 2015 (N = 280)

Characteristics	N (%)
Age in years, mean ± SD	55.05 ± 12.66
Below 35	10 (3.6)
35–44	47 (16.8)
45–54	76 (27.1)
55–64	76 (27.1)
65 and above	71 (25.4)
Gender	
Male	149 (53.2)
Female	131 (46.8)
Marital status	
Single/widowed	19 (6.8)
Married	220 (78.6)
Divorced	41 (14.6)
Educational level	
No formal education	108 (38.6)
Primary education (grade 1–8)	66 (23.6)
Secondary education (grade 9–12)	45 (16.1)
Tertiary education (diploma and/or above)	61 (21.7)
Religion	
Islam	130 (46.4)
Orthodox Christian	125 (44.6)
Protestant	25 (8.9)
Occupation	
Civil servant	78 (27.9)
Merchant	35 (12.5)
Farmer	46 (16.4)
Unemployed	121 (43.2)
Duration of hypertension in years, mean ± SD	5.1 ± 4.1 years
< 5	162 (57.9)
≥ 5	118 (42.1)
Type of comorbidity	
Diabetes mellitus	73 (26.1%)
Peripheral neuropathy	65 (23.2%)
Dyspepsia	32 (11.4%)
Hypertrophic heart disease	14 (5.0%)
Heart failure	7 (2.5%)
Chronic kidney disease	6 (2.1%)
Urinary tract infection	6 (2.1)
Human immune virus	4 (1.4)
Ischemic heart disease	4 (1.4)
Asthma	3 (1.1)
Sexual dysfunction	3 (1.1)
Others	9 (3.1)

**Fig. 2:**
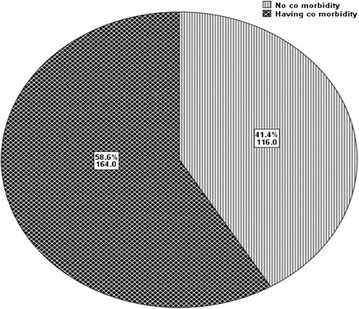
Prevalence of co morbidity among hypertensive patients at Jimma University Specialized Hospital from March 4, 2015 to April 3, 2015

On the other hand, more than half (n = 164, 58.6%) of the participants had at least one written evidence of co morbidity (Fig. 2). 73 (26.1%) participants had diabetes compared to 65 (23.2%) patients who had peripheral neuropathy (Table 1).

As depicted in Table 3, 173 (61.8%) of the participants were adherent to their antihypertensive medication compared to 107 (38.2%) patients who were non adherent (Table 2).

**Table 2:**
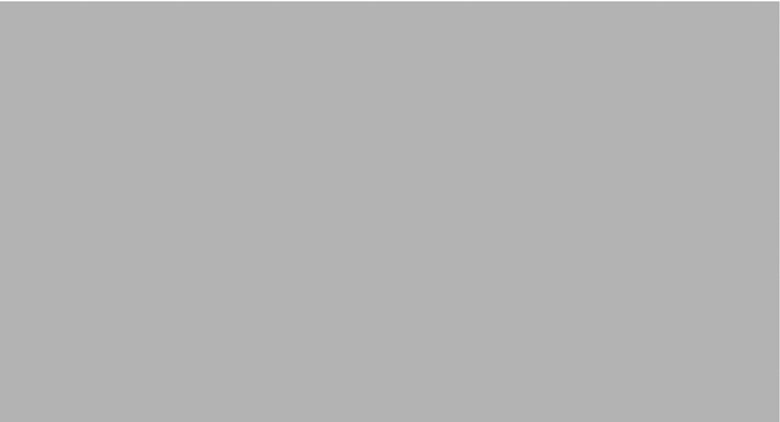
A summary of responses to the eight-item Moriskiy’s Medication Adherence Scale among adult hypertensive patients at Jimma University Specialized Hospital March 4, 2015 to April 3, 2015. This table been removed because the authors have not obtained a licence to use the Morisky Medication Adherence Scale-8 (MMAS-8). The results presented in this table are available by contacting the authors

### Antihypertensive medications and blood pressure control

**Fig. 3:**
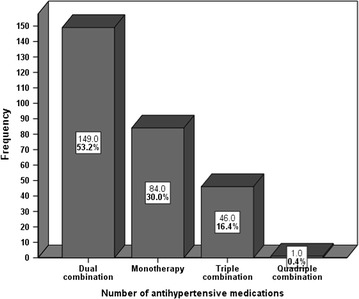
Number of antihypertensive medications among hypertensive patients at Jimma University Specialized Hospital March 4, 2015 to April 3, 2015

The average number of antihypertensive medications prescribed per patient was 2.9 ± 0.7. Among the 280 participants, 173 (61.8%) were prescribed with more than one antihypertensive medication. Above half (53.2%) of the study patients were on two medications compared to 84 (30%) patients who were on mono-therapy and 46 (16.4%) patients who were on triple therapy (Fig. 3). Among the participants on mono-therapy, 53 (63.1%) of them had controlled blood pressure.

The mean of SBP means of the participants was 132.5 ± 20.3 mmHg, range 90–190 mmHg and the mean of DBP means was 81.7 ± 12.0 mmHg, range 50–90 mmHg.

### Antihypertensive medication adherence and associated factors

As shown in Table 3, the presence of co-morbidity, regular alcohol intake, self-purchasing of the medications and combinations of antihypertensive medications were significantly but inversely associated antihypertensive medication adherence. Accordingly, patients with comorbidities (AOR = 0.083, CI = 0.033–0.207, p < 0.001) were 12 times more likely to be non-adherent compared to patients without comorbidities. As well, those taking alcohol (AOR = 0.011, 95% CI = 0.002–0.079, p < 0.001) were 90 times more likely to be non-adherent compared to patient who were not take alcohol. In addition, those paying for their medication (AOR = 0.020, CI = 0.003–0.117, p < 0.001) were 50 times more likely to be non-adherent, and those taking more than one drug (AOR = 0.32, 95% CI = 0.144–0.712, p < 0.005) were 3 times more likely to be non-adherent (Table 3).

**Table 3:** Results of logistic regression analysis for factors associated with medication adherence among adult hypertensive patients on treatment at Jimma University Specialized Hospital from March 4, 2015 to April 3, 2015

Variables	Medication adherence		COR (95% CI), *p*-value	AOR (95% CI), *p*-value
	Non adherent (%)	Adherent (%)		
Number of anti-HTN medications				
1 medication	24 (22.4)	83 (77.6)	(1)	(1)
≥ 2 medications	84 (48.6)	89 (51.4)	0.306 (0.178–0.527), *p* < 0.001*	0.32 (0.144–0.712*), *p* < 0.005*
Alcohol intake				
Yes	46 (95.8)	2 (4.2)	0.016 (0.004–0.067), *p* < 0.001*	0.011 (0.002–0.079), *p* < 0.001*
No (1)	62 (26.7)	170 (73.3)	(1)	(1)
Presence of co-morbidities				
Yes	92 (56.1)	72 (43.9)	0.125 (0.068–0.231), *p* < 0.001*	0.083 (0.033–0.207), *p* < 0.001*
No (1)	16 (13.8)	100 (86.1)	(1)	(1)
Obtaining of anti-HTN medications				
Paid	106 (56.1)	83 (43.9)	0.018 (0.004–0.074), *p* < 0.001*	0.020 (0.003–0.117), *p* < 0.001*
Free (1)	2 (2.2)	89 (97.8)		(1)

*AOR* adjusted odds ratio, *CI* confidence interval, *COR* crude odds ratio, *DM* diabetes mellitus, *HHD* hypertensive heart disease, *(1)* reference category, *HTN* hypertension

* Statistically significant at *p* < 0.05

## Discussions

Adherence to antihypertensive medications as measured using MMAS-8 in JUSH was 61.8% when defined by the MMAS-8 ≥ 6 cut off. Evidence of comorbidity, daily alcohol intake, purchasing medications with own expense and patients with two and more than two antihypertensive medications were the factors showing inverse association with medication adherence.

More than half, 61.8% of the study subjects in JUSH were found to be adherent to their antihypertensive medication treatment. This adherence level was lower than the findings of other studies reported in Egypt (77%), in Pakistan (77%) and in Scotland (91%) [26–28]. Variations in the studied populations, assessment tool and cut off points of the studies might contribute to the adherence variation between the studies. For instance, the study from Pakistan was used MMAS-4 as assessment tool to measure adherence [27]. Moreover, better health care and access of health facilities in the developing countries like Egypt and Scotland may also enhance patient’s adherence as compared to our study [26, 28]. On the other hand, the adherence level reported in this study was higher than the findings from Malaysia (44.2%) and Gambia (27%) [25, 26]. This difference might be explained as 32.2% of the participants in this study received free medical care and medications, whereas, in the aforementioned comparative studies, patients had to pay for their treatment. Our studies level of adherence was relatively quiet congruent to local studies conducted in Gonder, Northwest Ethiopia (64.6%) and Adama, Ethiopia (59.5%) [16, 17]. This might be owing to similarity in the study setting and economic status of the study participants.

In this study, co-morbidity was significantly associated with anti-hypertensive medication adherence. Study participants with one or more co-morbidities were less likely to adhere to their antihypertensive medications than their counter parts. There were also similar findings from Ethiopia and Saudi Arabia that showed comorbidity as factor tackling adherence [7, 16]. The plausible reason could be due to a complicated treatment regimen as a result of the increased number of medications prescribed for both the hypertension and the co-morbidities might result to polypharmacy and pill burden which ultimately hinder adherence.

Another important factor that influences patient compliance is the number of medications prescribed. Number of prescribed antihypertensive medications was also significantly associated with adherence. Participants who were taking two and/or more medications were less likely to adhere to their medications compared to participants who were on mono-therapy. There are similar findings from Greek [29] and Malaysia [26]. According to a systematic review of randomized controlled trials, reduction of the number of daily doses of antihypertensive medication, may improve adherence [30]. Regimens with one tablet per day are quite simple and as a consequence, patients find them easy to follow without forgetting their daily dose [31]. Hence combination and preparation of the two antihypertensive medication as one tablet might improve patient’s adherence. This is particularly true for the elderly hypertensive patient who usually suffer from several comorbidities. It must be noted that, it is difficult to adequately control blood pressure of elderly hypertensive patients and patients with comorbidity with a simple antihypertensive drug [32]. Thus, usually this problem can be solved with the use of a tablet, which combines more than one drug, which is very difficult to apply in our setting.

In addition to this, daily alcohol intake and purchasing medications with own expense were significantly associated with antihypertensive medication adherence. Daily alcohol intake significantly hinders adherence status of the participants. Nevertheless, this was not in line with a study done in Hyderabad [33]. This might be due to discrepancies in the assessment tool used. This study used MMAS-8 while the study from India used MMAS-4. Moreover, participants who purchased medications by themselves were less likely to adhere to their medications than participants who get their mediations freely. This is similar with a study done in northwest Ethiopia which showed higher odds of adherence to antihypertensive medications in respondents who had got the medication/s free of charge or with low cost as compared to those who had got the medication/s with high cost [34]. This might be attributable to the economic constraint of the former participants. Since Ethiopia is one of the developing countries low daily income in association with low awareness might hamper patient’s adherence. Thus, patient’s awareness creation and availing affordable medication might enhance adherence and ultimately improve patient’s outcome.

The study has certain limitation. A cross-sectional study was conducted which did not allow follow-up of the participants. Besides this, the study was conducted at a single center with a modest number of individuals, which might limit generalization of the findings to wider contexts.

## Conclusion

The adherence level to the prescribed antihypertensive medications was found to be sub-optimal. Moreover, daily alcohol intake, co-morbidity, number of antihypertensive medications and availability of medications without fee are the factors affecting adherence. These findings open multiple avenues that health care providers should pay due attention to the importance of adherence and the influencing factors of adherence.

## Additional file

**Additional file 1.** Morisky Medication Adherence Scales: MMAS-8. This file has been removed because the authors have not obtained a licence to use the Morisky Medication Adherence Scale-8 (MMAS-8).

## Abbreviations

AOR: adjusted odds ratio
BP: blood pressure
CI: confidence interval
COR: crude odds ratio
DASH: dietary approach to stop hypertension
DBP: diastolic blood pressure
JUSH: Jimma University Specialized Hospital
MMAS-8: Morisk’s medication adherence scale-8
SBP: systolic blood pressure
SPSS: statistical package for social science
WHO: World Health Organization
