# Correction to: Antihypertensive medication adherence and associated factors among adult hypertensive patients at Jimma University Specialized Hospital, southwest Ethiopia

**DOI:** 10.1186/s13104-018-3674-1

**Published:** 2018-08-16

**Authors:** Solomon Weldegebreal Asgedom, Tesfay Mehari Atey, Tigestu Alemu Desse

**Affiliations:** 1 School of Pharmacy, College of Health Sciences, Mekelle University, Mekelle, Ethiopia; 2 Department of Pharmacology and Clinical Pharmacy, School of Pharmacy, College of Health Sciences, Addis Ababa University, Addis Ababa, Ethiopia

## Correction to: BMC Res Notes (2018) 11:27 10.1186/s13104-018-3139-6

After publication of our article [1] we became aware that we had not obtained permission to reproduce the questions from the eight-item Morisky’s Medication Adherence Scale. The table in this Correction replaces Table 2 in our article. In addition, the second number for the MMAS-8 question 2 was incorrect and has been updated to 223 (79.6).

**Table 2:** A summary of responses to the eight-item Morisky’s Medication Adherence Scale [24] among adult hypertensive patients at Jimma University Specialized Hospital March 4, 2015 to April 3, 2015

MMAS-8 measurement scale questions	Frequency (%)	
	Yes	No
MMAS-8 question 1	89 (31.8)	191 (68.2)
MMAS-8 question 2	57 (20.4)	223 (79.6)
MMAS-8 question 3	27 (9.6)	253 (90.4)
MMAS-8 question 4	93 (33.2)	187 (66.8)
MMAS-8 question 5	242 (86.4)	38 (13.6)
MMAS-8 question 6	35 (12.5)	245 (87.5)
MMAS-8 question 7	141 (50.4)	139 (49.6)
MMAS-8 question 8		
Never/rarely	112 (40.0)	168 (60.0)
Once in a while	39 (13.9)	241 (86.1)
Sometimes	122 (43.6)	158 (56.4)
Usually	7 (2.5)	273 (97.5)
All the time	0 (0.0)	280 (100.0)

Adherence	Frequency (%)
Adherent	173 (61.8)
Non adherent	107 (38.2)

The reference which is cited in the above table caption can be reviewed in our article.
