# Supplementary material for: Antihypertensive medication adherence and associated factors among adult hypertensive patients at Jimma University Specialized Hospital, southwest Ethiopia
Source: BMC Res Notes. 2018 Jan 15;11:27. doi: 10.1186/s13104-018-3139-6 (PMC5769214; doi:10.1186/s13104-018-3139-6)
Supplement: Supplementary file 1 — Additional file 1. Morisky Medication Adherence Scales: MMAS-8. This file has been removed because the authors have not obtained a licence to use the Morisky Medication Adherence Scale-8 (MMAS-8). [file 13104_2018_3139_MOESM1_ESM.docx]

**Morisky Medication Adherence Scales: MMAS-8. This file has been removed because the authors have not obtained a licence to use the Morisky Medication Adherence Scale-8 (MMAS-8).**
